# Epidemiological study of congenital heart diseases in dogs: Prevalence, popularity, and volatility throughout twenty years of clinical practice

**DOI:** 10.1371/journal.pone.0230160

**Published:** 2020-07-27

**Authors:** Paola Giuseppina Brambilla, Michele Polli, Danitza Pradelli, Melissa Papa, Rita Rizzi, Mara Bagardi, Claudio Bussadori

**Affiliations:** 1 Department of Veterinary Medicine, University of Milan, Lodi, Italy; 2 Department of Cardiology, Clinica Veterinaria Gran Sasso, Milan, Italy; Scuola Superiore Sant'Anna, ITALY

## Abstract

The epidemiology of Congenital Heart Diseases (CHD_s_) has changed over the past twenty years. This study aimed to evaluate the prevalence of CHD_s_ in the population of dogs recruited in a single referral center (RC); compare the epidemiological features of CHD_s_ in screened breeds (Boxers) versus non-screened (French and English Bulldogs, German Shepherds); investigate the association of breeds with the prevalence of CHD_s_; determine the popularity and volatility of breeds over a 20-year period; analysed the trends of the most popular breeds in the overall population of new-born dogs registered in the Italian Kennel Club (IKC) from 1st January 1997 to 31st December 2017. The RC’s cardiological database was analysed, and 1,779 clinical records were included in a retrospective observation study. Descriptive statistics and frequencies regarding the most representative breeds and CHD_s_ were generated. A logistic regression model was used to analyse the trends of the most common CHD_s_ found in single and in cluster of breeds. The relationship between breed popularity and presence of CHD_s_ was studied. The most common CHD_s_ were Pulmonic Stenosis, Patent Ductus Arteriosus, Subaortic Stenosis, Ventricular Septal Defect, Aortic Stenosis, Tricuspid Dysplasia, Atrial Septal Defect, Double Chamber Right Ventricle, Mitral Dysplasia, and others less frequent. The most represented pure breeds were Boxer, German Shepherd, French Bulldog, English Bulldog, Maltese, Newfoundland, Rottweiler, Golden Retriever, Chihuahua, and others in lower percentage. Chihuahuas, American Staffordshire Terriers, Border Collies, French Bulldogs, and Cavalier King Charles Spaniel were the most appreciated all of which showed a high value of volatility. This study found evidence for the value of the screening program implemented in Boxers; fashions and trends influence dog owners’ choices more than the worries of health problems in a breed. Effective breeding programs are needed in order to control the diffusion of CHD_s_ without impoverishing the genetic pool.

## Introduction

Congenital anomalies of the cardiovascular system are defects present at birth, and often lead to perinatal death in dogs. However, in some cases, congenital heart diseases are asymptomatic and undetected until later in life, so the percentage of dogs with congenital heart diseases that survive to adulthood to breed can be rather high [1–7]. To decrease the incidence of CHD_s_ in the dog population as a whole, the early identification of affected dogs could inform a breeding program. Furthermore, some of the most common CHD_s_ could be successfully treated by surgical management, and an early diagnosis can help to provide a normal life expectancy compared to that of the untreated dogs [8]. Knowing the epidemiology of CHD_s_ plays an important role in maintaining dog health and in preventing the diffusion of CHD_s_ in the dog population [6].

Epidemiological studies on congenital heart disease in dogs have been conducted all over the world since the early 1960s [9,10].

The most valuable studies were performed in the USA, Australia, the UK, Switzerland, Sweden, Poland and Italy [11–16]. The main studies report different prevalence of CHDs in the affected breeds, depending on the popularity of the breed in a country in a given period of time [17–19]. In almost all studies, the most common CHD_s_ observed were Patent Ductus Arteriosus (PDA), Pulmonic Stenosis (PS), and Subaortic Stenosis (SAS) [8–16]. Ventricular Septal Defect (VSD), Tricuspid Dysplasia (TD) and Tetralogy of Fallot (TOF) have also been described by the authors of these studies, but they are not noted as frequently as the abovementioned CHD_s_ [6,9,10,12,14,15].

In 2011, a retrospective epidemiological study on CHD_s_ was performed in Italy by Oliveira et al. [16]. The included data were collected at a single veterinarian referral center for cardiovascular disease in small animals, specializing in the surgical and interventional treatment of congenital heart diseases [16]. Since 1997, the RC, Italian Boxer Club (IBC) and Fondazione Salute Animale (FSA) have also been included in a screening program that aims to reduce the prevalence of PS and SAS in Boxers, such that many breeding dogs have been screened for these conditions before breeding.

In the last seven years, new CHD_s_ clinical cases have been reported, and this phenomenon provided a worthy opportunity to evaluate the epidemiology of CHD_s_ in a large population of dogs in the same RC over a longer period of time.

The clinic’s database was updated and reanalyzed in order to: 1) assess the prevalence of CHD_s_ in the population of dogs recruited in a single RC, 2) investigate any changes in the epidemiological features of congenital heart diseases in non-screened (French Bulldog, English Bulldog and German Shepherd) and screened (Boxer) breeds, 3) determine the association of breeds with the prevalence of CHD_s_, 4) study the popularity and volatility of the breeds over this 20-year period. Finally, the trends of the most popular dog breeds in the overall population of the puppies registered in the IKC database from 1st January 1997 to 31st December 2017 were analyzed [20].

## Materials and methods

The medical records of dogs referred for congenital heart disorders to Clinica Veterinaria Gran Sasso between 1^st^ January 1997 to 31^st^ December 2017 were retrospectively reviewed.

The population affected by CHDs was organized in two spreadsheets (isolated and associated). Dogs affected by one CHD were included in the isolated Congenital Heart Disease group (CHD_i_), and dogs with two or more concurrent defects were included in the associated Congenital Heart Disease group (CHD_a_). In this study, TOF was included in the CHD_i_ group, because the pathology was considered as a unique entity.

The breeds with more than 20 dogs each and the defects diagnosed in more than 10 subjects were included. The breeds with fewer than 20 dogs and the CHD_s_ with fewer than 10 animals each were named as “others”.

Subaortic Stenosis Type 1, Type 2 and Type 3 were pooled in the SAS category, whereas Pulmonic Stenosistype A, B, M, B-hourglasses (BHG), M-hourglasses (MHG) and PS Anomalous Right Coronary Artery (R2ACA) were included in the PS group [21–30].

The inclusion criteria were pure breed dogs affected by CHD_s_ with complete clinical records (signalment, history, and physical examination), including thoracic radiography and echocardiography without sedation. The angiography and postmortem examination were not executed in all cases. The diagnosis of CHD was obtained by a complete transthoracic echocardiographic examination (TTE), which was performed in all patients. TTEs were carried out using commercial ultrasound equipment with mechanical transducers ranging from 2 to 10 MHz (Caris, Esaote, Florence, Italy), and then using ultrasound machines with electronic transducers also ranging from 2 to 10 MHz (Megas Esaote, Florence, Italy; Mylab30Vet, Esaote, Florence, Italy; MyLab60, Esaote, Florence, Italy, Epiq 7 Philips S.p.A., Milan, Italy).

Two-dimensional transesophageal echocardiography (TEE 2D) was executed using an omniplane transesophageal probe (Mylab30Vet, Esaote, Florence, Italy) ranging from 3 to 8 MHz. Three-dimensional TEEs were performed with an echocardiography machine equipped with an omniplane transesophageal probe x7 matrix ranging from 2 to 7 MHz (Philips IE33, S.p.A., Milan, Italy) when indicated and authorized by the owner.

The exams were performed, interpreted and/or reviewed by an ECVIM board-certified cardiologist (C.B.). The patients were placed in right and left lateral recumbency, and the examinations were performed according to the American Society of Echocardiography standards and guidelines and other published recommendations [31].

Angiographic procedures were also performed and/or reviewed by an ECVIM board-certified cardiologist (C.B.) with a fluoroscopy system in cases undergoing interventional percutaneous procedures or when necessary for diagnostic purposes (Villa Sistemi Medicali S.p.A., Buccinasco (MI), Italy and Digital Fluoroscopy system Philips Veradius, Milan, Italy). Postmortem examinations were performed under the supervision of C.B.

The crossbreed dogs were included as a single group, because the phenotypic features and the weight at presentation were unknown in some cases. This missing information did not allow a categorization into small, medium and large breeds.

The exclusion criteria were pure breed dogs with incomplete clinical records or all dogs that were affected by acquired heart disease.

The prevalence, the popularity and the volatility of the most common breeds were evaluated by using the cohort of the puppies registered in IKC database from January 1997 to December 2017.

### Statistical analysis

Two datasets were available for statistical analyses, and the descriptive statistics were calculated. The Kolmogorov-Smirnov test was used to assess normality of the continuous variables. For data that were not normally distributed, median and interquartile ranges (I/Q: lower and upper quartiles) are given. The Kruskal-Wallis test was used to evaluate the significance of the association among the most common CHD_s_ and the age at presentation. The frequency distributions of all diseases in the overall sample for sex was calculated. The χ^2^ test was used to compare the occurrence of each CHD in males and females both of purebreeds and crossbreeds.

The combinations of the observed heart defects were analyzed as groups of two, three, four and five CHDs, except TOF, which was considered as a unique entity.

The changes in the epidemiological features were investigated for the CHDs and the breeds with a suitable number for statistical analyses. Therefore, the trend of PS from 1997 to 2017 was evaluated by comparing the Boxer breed both with two brachycephalic breeds (English Bulldog, French Bulldog) and with the large size breeds (German Shepherd, Newfoundland, Rottweiler, Golden retriever, Labrador retriever, American Staffordshire, Dobermann). Moreover, a comparison between the trends of PDA throughout the same period was carried out in the German Shepherd and in the large size breeds (Newfoundland, Rottweiler, Golden retriever, Labrador retriever, American Staffordshire, Dobermann). For this analysis, the years were pooled in 7 periods for a better description of the CHD_s_ trend: 1997–1999, 2000–2002, 2003–2005, 2006–2008, 2009–2011, 2012–2014, and 2015–2017. For each CHD, dogs were considered ‘positive’ or ‘negative’ if they were affected or were not affected by that CHD, respectively. The risk of finding dogs with a determined CHD in a specific period and for a specific breed can be estimated by the following generalized linear model

E[Y|γ]=Xβ+ε

where Y is the vector of observations, β is the vector of the fixed effect (breed * period interaction) and var[ε] = var[Y|γ]. This model, applied to a binomial distribution, provides the least square means and the relative confidence intervals on a logit scale; the least square means can be reported to the probability scale by the following equation:

$$logit(p)=\ln\left(\frac{p}{1-p}\right)=ln(p)-ln(p-1)$$

This equation can be rearranged as:

$$p=\frac{e^{logit}}{1+e^{logit}}$$

The Clinica Gran Sasso internal CHD_s_ database was merged with the IKC database in order to estimate the odds ratios of the overall CHD_s_. Contingency tables were constructed for the relationships between overall CHD_s_ and each breed. The magnitude of the relationship was expressed as the odds ratio and relative 95% CI with associated P-value.

To investigate the association of CHD_s_ and the breeds’ popularity, the Pearson correlation coefficients were estimated for the total number of CHD_s_ detected in each breed, the OR of overall CHD were determined, and two measures of breed popularity were calculated as reported by Ghirlanda et al. [18]:

1. Total popularity, defined as the total number of registrations for each breed in 1997–2017:
  $$t_{i}=\sum_{k=1997}^{2017}p_{i,k}$$
2. Volatility, defined as the average relative change in registrations from one year to the next:
  $$v_{i}=\frac{1}{21}\sum_{k=1997}^{2017}\left|\frac{p_{i,k}-p_{i,k-1}}{p_{i,k}}\right|$$
  where:
  t_i_ = popularity of the i^th^ breed;
  v_i_ = volatility of the i^th^ breed;
  p_i,k_ = number of dogs of the i^th^ breed registered in IKC in a year k (1997≤k≤2017);
  21 = the number of registration changes in the period 1997–2017.

For all analyses, statistical significance was set to the 5% level.

Statistical analyses were performed using the GLIMMIX, FREQ, MEANS and UNIVARIATE SAS^®^ procedures (SAS Institute Inc. Base SAS^®^ 9.4 Procedures Guide: Statistical Procedures, Second Edition. Cary, NC: SAS Institute Inc. 2013).

## Results

This retrospective study was based on the 1,779 clinical records that fulfilled the inclusion criteria. Single cardiac defects were present in 1,568 dogs (88.14%), and 2 or more concurrent defects were found in 211 dogs (11.86%). The total observed cases of congenital heart defects are reported in Table 1, including information on sex at presentation.

**Table 1:** Summary of congenital heart defects of the included population.

CHDs	TOTAL		CHD_i_		CHD_a_		MALE				FEMALE				AGE (months)
	N	%	N	%	N	%	CHD_i_	CHD_a_	TOTAL	%	CHD_i_	CHD_a_	TOTAL	%	Q_2_ (Q_1_ –Q_3_)
**PS**	689	34.1	570	82.7	119	17.3	339	67	406	58.9	231	52	283	41.1	10 (5–24)
**PDA**	534	26.4	490	71.1	44	8.2	156	15	171	32.0	334	29	363	68.0	7 (3–20.5)
**SAS**	296	14.6	220	31.9	76	25.7	139	44	183	61.8	81	32	113	38.2	12.5 (4 31)
**VSD**	98	4.8	39	5.7	59	60.2	21	33	54	55.1	18	26	44	44.9	8 (4–20.5)
**AS**	95	4.7	80	11.6	15	15.8	54	7	61	64.2	26	8	34	35.8	25 (10–81)
**TD**	69	3.4	51	7.4	18	26.1	26	8	34	49.3	25	10	35	50.7	10 (6–28)
**ASD**	42	2.1	21	3.0	21	50.0	10	3	13	31.0	11	18	29	69.0	15 (7–35)
**DCRV**	37	1.8	21	3.0	16	43.2	14	9	23	62.2	7	7	14	37.8	6 (4–11)
**MD**	32	1.6	27	3.9	5	15.6	15	5	20	62.5	12	0	12	37.5	8 (4–17.5)
**TOF**	21	1.0	0	0.0	21	100.0	0	11	11	52.4	0	10	10	47.6	5 (3–9)
**rPDA**	15	0.7	15	2.2	0	0.0	6	0	6	40.0	9	0	9	60.0	11 (5–29)
**MVS**	12	0.6	6	0.9	6	50.0	4	4	8	66.7	2	2	4	33.3	26.5 (3–76)
**BAV**	10	0.5	5	0.7	5	50.0	3	5	8	80.0	2	0	2	20.0	14 (2–26)
**PLCVC**	10	0.5	0	0.0	10	100.0	0	6	6	60.0	0	4	4	40.0	6.5 (4–21)
**AVCD**	10	0.5	5	0.7	5	50.0	0	1	1	10.0	5	4	9	90.0	14 (10–47)
**others**^**a**^	52	2.6	18	2.6	34	65.4	6	14	20	38.5	12	20	32		10 (4–54)
	2,022	100	1,568		454		793	232	1,025		775	222	997		

CHD_s_, congenital heart diseases; CHD_i_, isolated congenital heart diseases; CHD_a_, associated congenital heart diseases; PS, pulmonic stenosis; PDA, Patent Ductus Arteriosus; SAS, Subaortic Stenosis; VSD, Ventricular Septal Defect; AS, aortic stenosis; TD, Tricuspid Dysplasia; ASD, Atrial Septal Defect; DCRV, Double Chamber Right Ventricle; MD, Mitral valve Dysplasia; TOF, Tetralogy of Fallot; rPDA, reverse Patent Ductus Arteriosus; MVS, Mitral valve Stenosis; BAV, Aortic Bicuspid Valve; PLCVC, persistent left cranial vena cava; AVCD, Atrioventricular Canal Disease

others^a^ CTD, Cor Triatriatum Dexter; AVF, Aortovenous Fistula; PPDH, Pericardial Peritoneum Diaphragmatic Hernia; QAV, Quadricuspid Aortic Valve; AH, Aortic Hypoplasia; AI, Aortic Insufficiency; AOr, Aortic Overriding; APW, Aortopulmonary Window; MI, Mitral Insufficiency; TVS, Tricuspid Valve Stenosis; TA, Truncus Arteriosus; CollF, Systemic to Pulmonary Arterial Collateral Flow; AC, Azygos Continuation; ACT, Anomalous Coronary Truncus.

The most common CHD in our overall population, both isolated and associated with other conditions, were PS (34.1%), PDA (26.4%) SAS (14.6%), VSD (4.8%), AS (4.7%), TD (3.4%), ASD (2.1%), DCRV (1.8%), MD (1.6%), TOF (1%) and rPDA (0.7%) (Table 1).

**Fig 1:**
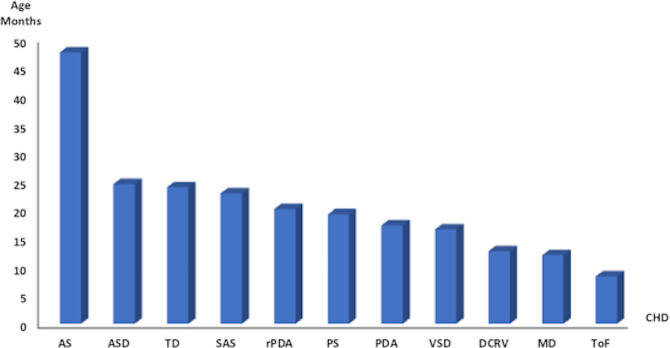
Average age (months) of the pure breed dogs belonging to the most represented isolated CHD_s_. AS, Aortic Stenosis; ASD, Atrial Septal Defect; TD, Tricuspid Dysplasia; SAS, Subaortic Stenosis; rPDA, Reverse Patent Ductus Arteriosus; PS, Pulmonary Stenosis; PDA, Patent Ductus Arteriosus; VSD, Ventricular Septal Defect; DCRV, Double Chamber Right Ventricle; MD, Mitral Dysplasia; TOF, Tetralogy of Fallot.

Among the most common CHD_s_ in pure breeds, the youngest dogs at presentation were affected by TOF (median 5 month) and the oldest by AS (median 25 months) (Fig 1). The results from the Kruskall-Wallis test showed that the dogs diagnosed with AS were significantly older than the dogs affected by PDA, PS, DCRV, VSD, SAS, TD, and TOF (Fig 1).

Isolated congenital heart diseases were diagnosed in 1,377 dogs belonging to 92 purebreeds and 191 crossbreeds. The top 21 represented purebreeds were Boxer (19.4%), German Shepherd (9.4%), French Bulldog (6.2%), English Bulldog (4.9%), Maltese (3.7%), Newfoundland (3.1%), Rottweiler (3.1%), Golden Retriever (3.0%), Chihuahua (2.8%), Poodle (2.5%), Labrador Retriever (2.3%), Cavalier King Charles Spaniel (2.2%), American Staffordshire Terrier (2.1%), Dobermann (2.1%), Miniature Pinscher (2.0%), Cocker Spaniel (2.0%), Yorkshire Terrier (1.7%), Border Collie (1.6%), Dachshund (1.6%), Dogue de Bordeaux (1.6%), and Bull Terrier (1.5%).

The most common CHD in this study proved to be PS, as a single or complex defect, associated with SAS (22.29%), VSD (19.11%), PDA (8.28%) and less common CHD_s_ (9.55%) (Tables 1 and 2).

**Table 2:** Summary of the percentage distribution of CHD_s_ in purebreeds and in crossbreeds included in this study.

Breed	PS	PDA	SAS	AS	TD	VSD	MD	DCRV	ASD	rPDA	other CHD_s_ ^a^
**Boxer**	34.8	-	37.5	19.9	3.0	0.4	0.4	0.4	2.6	-	1.1
**German shepherd**	8.5	65.9	14.0	3.1	4.7	-	-	-	0.8	-	3.1
**French Bulldog**	82.6	4.7	-	3.5	-	7.0	1.2	1.2	-	-	-
**English Bulldog**	88.1	1.5	1.5	1.5	4.5	-	-	-	-	-	3.0
**Maltese dog**	5.9	76.5	-	-	-	5.9	2.0	-	-	9.8	-
**Newfoundland**	11.9	42.9	38.1	-	4.8	-	-	-	-	-	2.4
**Rottweiler**	23.8	7.1	45.2	2.4	2.4	-	4.8	2.4	2.4	-	9.5
**Golden retriever**	29.3	7.3	31.7	2.4	14.6	-	4.9	9.8	-	-	-
**Chihuahua**	25.6	59.0	-	-	-	2.6	-	10.3	-	2.6	-
**Poodle**	20.0	65.7	-	-	5.7	2.9	-	-	2.9	2.9	-
**Labrador retriever**	6.5	22.6	12.9	-	35.5	3.2	6.5	-	3.2	3.2	6.5
**Cavalier King Charles**	40.0	60.0	-	-	-	-	-	-	-	-	-
**American Staffordshire**	86.2	-	-	3.5	-	-	6.9	3.5	-	-	-
**Dobermann**	3.5	89.7	-	-	-	-	-	-	3.5	-	3.5
**Miniature Pinscher**	96.4	3.6	-	-	-	-	-	-	-	-	-
**Cocker Spaniel**	59.3	37.0	-	-	-	-	-	-	-	-	3.7
**Yorkshire terrier**	30.4	47.8	-	-	-	8.7	4.4	-	4.4	-	4.4
**Border Collie**	4.6	59.1	-	-	-	18.2	-	-	4.6	-	13.6
**Dachshund**	9.1	68.2	4.6	-	-	4.6	4.6	-	-	-	9.1
**Dogue de Bordeaux**	4.6	-	72.7	4.6	9.1	-	-	-	-	-	9.1
**Bull Terrier**	-	5.0	15.0	30.0	5.0	-	30.0	5.0	-	-	10.0
**other purebreeds**^**b**^	39.3	36.3	7.5	3.1	2.4	4.1	2.0	2.0	1.0	1.4	1.0
**Crossbreeds**	41.4	42.9	2.6	1.1	1.1	3.7	1.1	1.1	1.1	1.6	2.6

CHDs, congenital heart diseases; PS, pulmonic stenosis; PDA, Patent Ductus Arteriosus; SAS, sub-aortic stenosis; AS, aortic stenosis; TD, Tricuspid Dysplasia; VSD, Ventricular Septal Defect; MD, Mitral valve Dysplasia; DCRV, Double Chamber Right Ventricle; ASD, Atrial Septal Defect; rPDA, reverse Patent Ductus Arteriosus

^a^ MVS, Mitral valve Stenosis; BAV, Aortic Bicuspid Valve; AVCD, Atrioventricular Canal Disease; CTD, Cor Triatriatum Dexter; AVF, Aortovenous Fistula; PPDH, Pericardial Peritoneum Diaphragmatic Hernia; QAV, Quadricuspid Aortic Valve; AH, Aortic Hypoplasia; AI, Aortic Insufficiency; AOr, Aortic Overriding; APW, Aortopulmonary Window; MI, Mitral Insufficiency; TVS, Tricuspid Valve Stenosis; TA, Truncus Arteriosus; CollF, Systemic to Pulmonary Arterial Collateral Flow; AC, Azygos Continuation; ACT, Anomalous Coronary Truncus;.

^b^ West Highland white terrier; German Spitz; Corso Dog; Beagle; Jack Russel; Schnauzer; English setter; Pitbull; Australian Shepherd; Fox Terrier; Bernese mountain dog; Bolognese; Pembroke Welsh Corgi; Belgian shepherd; Greyhound; Shi tzu; Epagneul Breton; Pug; Swiss shepherd; Weimaraner; Akita Inu; Bichon Frisè; Bullmastiff; Great Dane; Italian Segugio; Shiba Inu; Czechoslovakian Wolfdog; Greater Swiss Mountain Dog; Neapolitan Mastiff; Pekingese dog; Pyrenean Mountain Dog; Basset hound; Bergamasco shepherd; Bobtail; Chow Chow; Dogo Argentino; Griffon Blue de Guascogne; Hungarian Bracco; Irish setter; Italian Greyhound; Italian shepherd dog; Lagotto; Maremma Sheepdog; Miniature Schnauzer; Pointer; Samoyed; Shetland Sheepdog; Welsh Terrier; Whippet; Alaskan Malamute; Appenzeller Mountain dog; Bavarian Mountain dog; Cairn Terrier; Cirneco dell'Etna; Dalmatian; Flat Coat retriever; Irish terrier; Italian Bracco; Jagd terrier; Karst shepherd; Lancashire Heeler; Magyar Agar; Norfolk terrier; Parson Russel terrier; Perro de Agua; Podenco ibicenco; Rhodesian Ridgeback; Scottish shepherd; Shar Pei; Spinone; Saint Bernard Dog.

**Fig 2:**
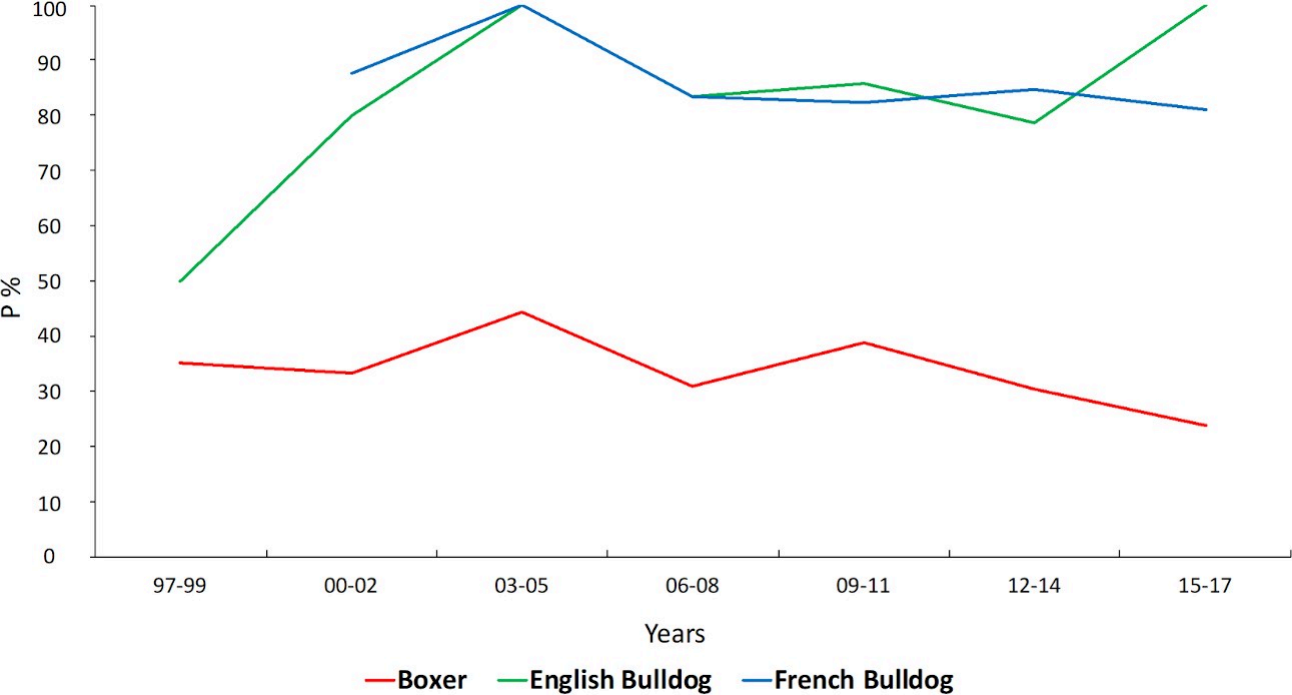
Probability of identifying Pulmonic Stenosis in Boxers, French Bulldogs and English Bulldogs. Red line: Boxer; Green line: English Bulldog; Blue line: French Bulldog.

The main breeds affected by all types of PS were brachycephalic breeds. English Bulldogs (88.1%) and French Bulldogs (82%) had the greatest prevalence of PS from the beginning of the study, and the prevalence increase over time as the Boxer PS prevalence decreased (Fig 2).

The most common PS Types found in the aforementioned breeds were Type A in French Bulldogs (42.25%), Type A equal to Type B in English Bulldogs (40.68%) and Type A in Boxers (59.14%).

In Boxers, only Types A and B of PS were found, while other Types were found in French Bulldogs (PS Type BHG 12.68%; PS Type M 15.49%) and in English Bulldogs (PS Type BHG, M and MHG, all together 18.64%). Although most common in English Bulldogs, PSR 2ACA was also found in French Bulldogs, Brussels Griffons, American Staffordshire Terriers and Corso dogs.

**Fig 3:**
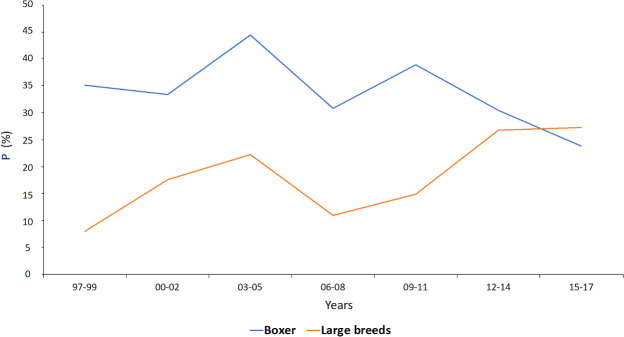
Probability of identifying Pulmonic Stenosis in Boxers and other large breeds admitted from 1997 to 2017. Blue line: Boxer; Orange line: other large breeds (American Staffordshire Terrier; Golden Retriever; German Shepherd; Rottweiler). P, Probability (%). X-axis: years.

Pulmonic Stenosis in American Staffordshire Terriers (86.2%) and Golden Retrievers (29.3%) progressively increased from 1997 and 2017 (Fig 3).

Interestingly, PS was also the most common CHD in Miniature Pinschers, in which the only other congenital heart disease observed was PDA (Table 2).

**Fig 4:**
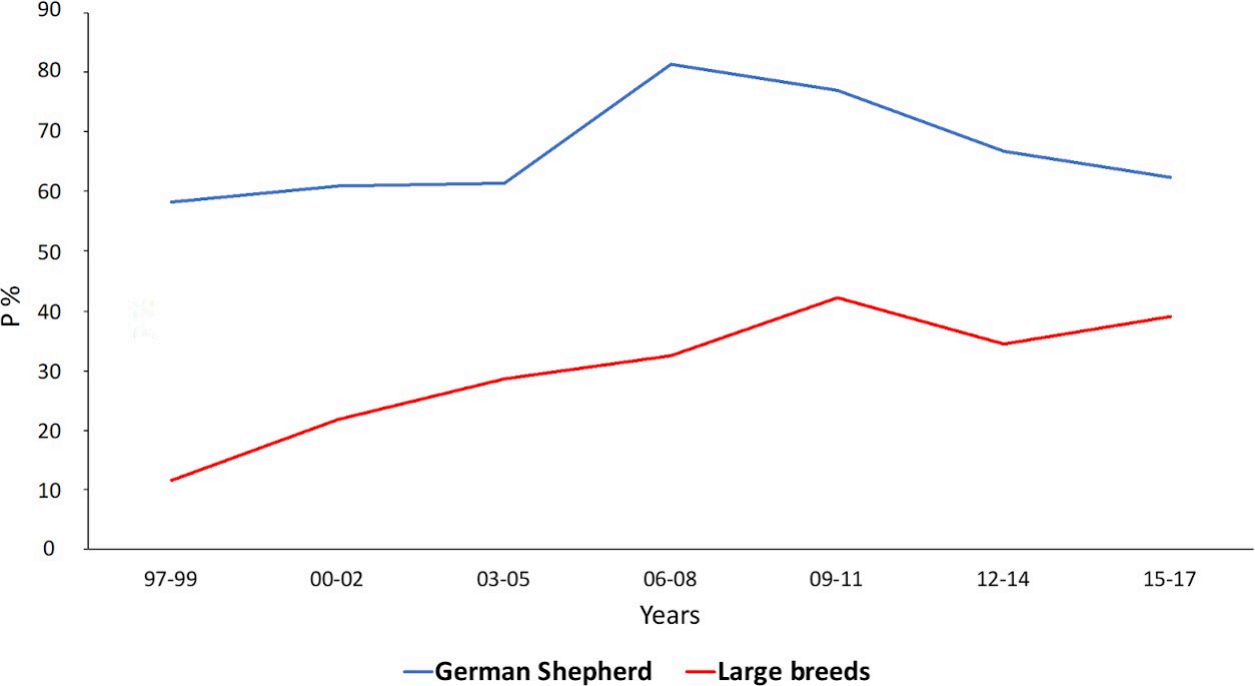
Probability of identifying Patent Ductus Arteriosus in large breeds vs German Shepherd admitted from 1997 to 2017. Blue line: German Shepherd; Red line: other large breeds (Bull Terrier; Dobermann; Golden Retriever; Labrador Retriever; Newfoundland; Rottweiler). P, Probability (%). X-axis: years.

PDA was the 2^nd^ most commonly observed CHD in our population, in both pure and crossbreed dogs; it was frequent in large dog breeds including Dobermanns (89.7%), German Shepherds (65.9%), and Newfoundland (42.9%), as well as in medium and small breeds such as Maltese (76.5%), Poodles (65.7%), CKCS (60%), Border Collies (59.1%) and Chihuahuas (59%). The highest frequency of PDA was observed from 2006 to 2011, and then decreased [12,13,16,32,33] (Fig 4). In our study population PDA was absent in Boxers, American Staffordshire Terriers and Dogue de Bordeaux. However, the same breeds experienced a large percentage of cases of PS (Boxer and American Staffordshire Terrier) and SAS (Dogue the Bordeaux) (Table 2). A small percentage of breeds experienced rPDA, and it was mostly detected in Maltese (9.8%) (Table 2). The presence of PDA was significant in females.

Up to 2011 SAS was the 2^nd^ most common defect in our study, and then was the 3^rd^, as a single defect or associated with PS or PDA (8.28%). SAS was found in 72.7% of the Dogue de Bordeaux admitted to the RC from 1997 to 2017, and Type 2 and Type 3 were the most frequent (36.36% each) (Tables 1,2 and 3).

**Table 3:** Summary of CHD_s_ associations in the included population.

CHD_a_		N	%
PS	SAS	35	22.29
PS	VSD	30	19.11
PS	PDA	13	8.28
PS	Other CHDs ^a^	15	9.55
SAS	PDA	13	8.28
SAS	VSD	5	3.18
SAS	other CHD_s_ ^b^	10	6.37
VSD	DCRV	7	4.46
VSD	other CHD_s_ ^c^	6	3.82
ASD	AVCD	5	3.18
other combinations ^d^		18	11.46

CHD_a_, associated congenital heart diseases; CHD_s_, congenital heart diseases, PS, pulmonic stenosis; VSD, Ventricular Septal Defect; PDA, Patent Ductus Arteriosus.

^a^ DCRV, Double Chamber Right Ventricle; AS, aortic stenosis; ASD, Atrial Septal Defect; TD, tricuspid valve dysplasia; AVF, aortovenosus fistula; PLCVC, persistent left atrial vena cava; TVS, Tricuspid Valve Stenosis.

^b^ TD, tricuspid valve dysplasia; MD, Mitral valve Dysplasia; MVS, Mitral valve Stenosis; BAV, bicuspid aortic valve; PLCVC, persistent left atrial vena cava; QAV, Quadricuspid Aortic Valve; TOF, Tetralogy of Fallot

^c^ PDA, Patent Ductus Arteriosus; ASD, Atrial Septal Defect; MD, Mitral valve Dysplasia; AOr, Aortic Overriding.

^d^ ASD-AS, Atrial Septal Defect–Aortic Stenosis; ASD-TD, Atrial Septal Defect–Tricuspid Dysplasia; ASD–AVCD, atrial septal defect-atrioventricular canal defects; PDA-AS, Patent Ductus Arteriosus—Aortic Stenosis; PDA–ASD, Patent Ductus Arteriosus-atrial septal defect; PDA–TD, Patent Ductus Arteriosus-Tricuspid Dysplasia; PDA–MVS, Patent Ductus Arteriosus-Mitral valve Stenosis; PDA–AVF, Patent Ductus Arteriosus- aortovenosus fistula; TD–DCRV, Tricuspid Dysplasia—Double Chamber Right Ventricle; TD–AS, Tricuspid Dysplasia–atrial septal defect TD–CTD, Tricuspid Dysplasia- Cor Triatriatum Dexter; TOF–APW, Tetralogy of Fallot-Aortopulmonary Window; AS–BAV, Aortic Stenosis-bicuspid aortic valve; DCRV—HCM; MVS–TVS, Mitral valve Stenosis- Tricuspid Valve Stenosis.

The three different subtypes were quite equally distributed in Boxers as Type 1 (39.6%), Type 2 (36.63%) and Type 3 (23.76%).

AS was the 4^th^ most common CHD in our population. AS was significantly more frequent in males (7.51% CI 4.57–7.05, P< 0.005) (Table 4) than in females, and Bull Terriers were the most affected breed (30%) (Table 2).

**Table 4:** Summary of the distribution of CHD_s_ by sex in purebreeds.

CHD_s_	N	Frequency	95% CI	Male frequency	Female frequency	p-value
**PS**	491	35.66	33.13–38.19	41.17	29.64	<0.0001
**PDA**	408	29.63	27.22–32.04	19.47	40.73	<0.0001
**SAS**	213	15.47	13.56–17.38	18.64	12.01	0.001
**AS**	80	5.81	4.57–7.05	7.51	3.95	0.005
**TD**	49	3.56	2.58–4.54	3.48	3.65	0.86
**VSD**	32	2.32	1.53–3.12	2.50	2.13	0.64
**MD**	25	1.82	1.11–2.52	1.95	1.67	0.70
**DCRV**	19	1.38	0.76–2.00	1.81	0.91	0.15
**ASD**	17	1.23	0.65–1.82	1.25	1.22	0.95
**rPDA**	12	0.87	0.38–1.36	0.56	1.22	0.19
**other CHD**_**s**_ ^**a**^	31	2.25	1.47–3.04	1.67	2.89	0. 48

CHDs, congenital heart diseases; PS, pulmonic stenosis; PDA; Patent Ductus Arteriosus; SAS, SubAortic Stenosis; AS, Aortic Stenosis; TD, Tricuspid Dysplasia; VSD, Ventricular Septal Defect; MD, mitral dysplasia; DCRV, Double Chamber Right Ventricle; ASD, atrial septal defect; rPDA, reverse Patent Ductus Arteriosus.

^a^ MVS, Mitral valve Stenosis; BAV, Aortic Bicuspid Valve; AVCD, Atrioventricular Canal Disease; CTD, Cor Triatriatum Dexter; AVF, Aortovenous Fistula; PPDH, Pericardial Peritoneum Diaphragmatic Hernia; QAV, Quadricuspid Aortic Valve; AH, Aortic Hypoplasia; AI, Aortic Insufficiency; AOr, Aortic Overriding; APW, Aortopulmonary Window; MI, Mitral Insufficiency; TVS, Tricuspid Valve Stenosis; TA, truncus arteriosus; CollF, Systemic to Pulmonary Arterial Collateral Flow; AC, Azygos Continuation; ACT, Anomalous Coronary Truncus.

In Tables 4 and 5, the frequency of the most common CHD_s_ by sex are reported for the purebreeds and crossbreeds, respectively.

**Table 5:** Summary of the distribution of the CHD_s_ by sex in crossbreeds.

CHD_s_	N	Frequency	95% CI	Male frequency	Female frequency	p-value
**PDA**	82	42.93	40.32–45.55	21.62	56.41	< .0001
**PS**	79	41.36	38.76–43.96	58.11	30.77	<0.0002
**other CHD**^**a**^	30	15.71	13.79–17.63	20.27	12.82	0.17

CHD_s_, congenital heart diseases; PDA, Patent Ductus Arteriosus; PS, Pulmonic Stenosis

^a^ VSD, Ventricular Septal Defect; SAS, Subaortic Stenosis; rPDA, reverse Patent Ductus Arteriosus; AS, Aortic Stenosis; ASD, Atrial Septal Defect; DCRV, Double Chamber Right Ventricle; MD, Mitral valve Dysplasia; TD, tricuspid valve dysplasia; AVCD, Atrioventricular Canal Disease; AVCDp, partial Atrioventricular Canal Disease; AVF, aortovenus fistula.

In purebreeds, PS, SAS and AS were significantly more frequent in males (p<0,005) while PDA was significantly more frequent in females (p<0,0001) (Table 4).

PS and PDA were also the most common cardiac defects in crossbreeds, and PDA was detected significantly more frequently in females, and PS was detected more frequently in males (Table 5).

**Fig 5:**
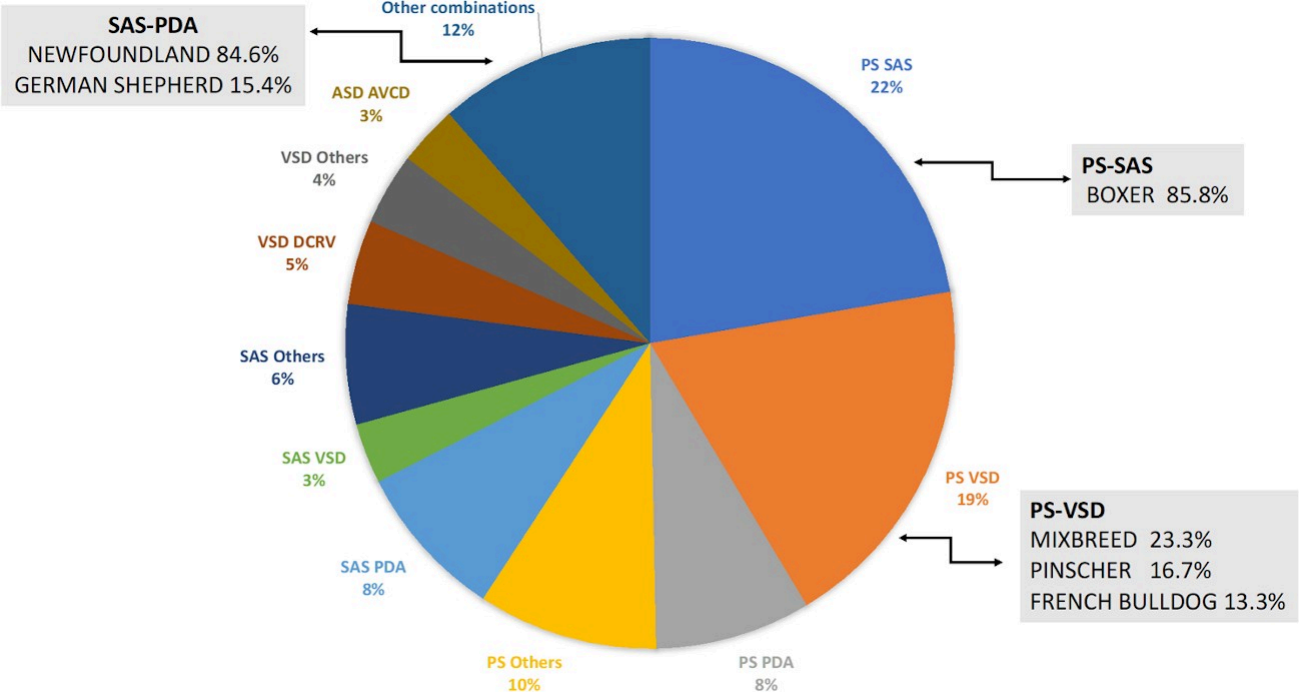
Associated congenital heart defects admitted from 1997 to 2017. PS, pulmonic stenosis; SAS, Subaortic Stenosis; VSD, Ventricular Septal Defect; PDA, Patent Ductus Arteriosus; DCRV, Double Chamber Right Ventricle; ASD, Atrial Septal Defect; AVCD, Atrioventricular Canal Disease.

In 189 purebreeds and in 22 crossbreeds, two or more defects were detected. The most frequent association was among two simple defects (74.4%), and PS was the most frequently detected disease (59.24%). PS was associated with SAS (22.29%), VSD (19.11%) and PDA (8.28%). SAS was associated with PDA in 8.28% of the dogs (Table 3, Fig 5).

There was 18% of dogs affected by a combination of three or more defects, and PS was the most frequently detected (65.8%).

The TOF was found in 21 dogs and in all of them was observed as isolated CHD condition (Table 1).

A significant and positive relationship between the overall number of CHD_s_ and popularity of breed was observed, suggesting that the prevalence of CHD_s_ grows as the number of IKC registered dogs increases (r = 0.54, p = 0.01).

The number of the CHD_i_ found in the selected breeds, the OR for the overall CHD_i_, the popularity of the breeds and the volatility for each breed are reported in Table 6.

**Table 6:** Summary of CHD_i_, OR referred to overall CHD (with 95% confidence interval), popularity, and volatility in each breed.

Breed	CHD_i_	OR	p value	t_i_	v_i_
**Chihuahua**	6	0.90 (0.65–1.24)	0.593	51457	13
**American Staffordshire**	6	0.89 (0.61–1.28)	0.6586	38859	11
**Border Collie**	7	0.70 (0.46–1.07)	0.120	36973	11
**French Bulldog**	10	6.98 (5.60–8.71)	0.000	15855	10
**Cavalier King Charles**	3	2.29 (1.59–3.29)	0.000	15904	9
**Golden retriever**	10	0.55 (0.40–0.75)	0.000	86696	7
**English Bulldog**	12	2.74 (2.14–3.51)	0.000	30417	6
**Labrador retriever**	10	0.22 (0.15–0.31)	0.000	154498	4
**Dachshund**	7	0.53 (0.34–0.80)	0.001	49035	4
**Miniature Pinscher**	5	5.60 (3.84–8.15)	0.000	6127	3
**Cocker Spaniel**	5	0.84 (0.57–1.23)	0.419	38247	2
**Dogue de Bordeaux**	7	2.02 (1.33–3.09)	0.003	13130	2
**Maltese dog**	5	2.30 (1.74–3.05)	0.000	27179	2
**Poodle**	6	1.18 (0.84–1.65)	0.352	35703	0
**Bull Terrier**	8	1.61 (1.03–2.50)	0.045	14974	-2
**German shepherd**	13	0.32 (0.26–0.38)	0.000	386970	-3
**Rottweiler**	15	0.52 (0.38–0.70)	0.000	93858	-4
**Boxer**	14	4.01 (3.50–4.61)	0.000	97670	-4
**Doberman**	4	0.66 (0.46–0.96)	0.029	51524	-7
**Newfoundland**	7	2.88 (2.11–3.92)	0.000	17924	-10
**Yorkshire terrier**	6	0.94 (0.62–1.42)	0.918	29099	-10

CHD_i_, isolated congenital heart diseases; t_i_, popularity; v_i_, volatility.

Chihuahuas, American Staffordshire Terriers, Border Collies, French Bulldogs and Cavalier King Charles Spaniels were the most popular small and medium breeds, and all of these breeds showed a high value of volatility (v_i_≥9) (Table 6). The probability of detecting a CHD_i_ at first presentation is significantly elevated in French Bulldogs, Cavalier King Charles Spaniels, English Bulldogs and Miniature Pinschers (Table 6). Large breeds, as Boxers and German Shepherds, on the contrary, demonstrated a decrease in volatility along the same period of time, even though the popularity of the breeds is higher than that found in small and medium breeds (Table 6).

## Discussion

To the best of our knowledge, this is the epidemiological study that included the highest number of dogs recruited in a single referral center over such a long period of time.

Since the clinic involved in this study is a RC for CHD_s_ in the authors’ country since 1997 and is located in a large city in the northern part of the country, it allows proper monitoring of the evolution of trends among different breeds over the years. In accordance with other studies, PS, PDA, SAS and AS were the most common CHD_s_ in the purebreeds population, PDA and PS were prevalent in crossbreeds, and in both groups, males were significantly more frequently affected by CHD_s_ than females [11–16,27]. There were, however, some differences concerning the prevalence of CHD_s_ in pure and mixed breeds [6,34].

The probability of admitting a Boxer affected by PS decreased from 1997 (35%) to 2017 (23.8%) in the overall population of the RC (Fig 2). This result can be explained as an effect of the screening program that has been in effect since 2000 in the RC. In collaboration with IBC and FSA, the screening program collected, in a separate database, the individual phenotypic information on the traits leading to a PS diagnosis, which then gradually led to a reduction of Boxers affected by this pathology [27,35]. The increased number of veterinary centers qualified to perform the screening before breeding Boxers could also be a reason to explain the reduction of incoming Boxers affected by PS in the authors’ RC.

In the last decade, English Bulldogs and French Bulldogs have been dramatically increasing in popularity in our country, as observed in this study’s population. Decreased popularity of Boxers was a trend that was observed in the results published by other authors and in our clinic, as mentioned above [17,36,37].

The factors that influenced the success of brachycephalic breeds are well known by authors in UK, Denmark and the USA, where many studies have been conducted [37,38]. The lovers of the brachycephalic breeds were less influenced by health and longevity in terms of breed selection compared with non-brachycephalic dogs’ owners [39,40]. A variety of different drivers have been identified to explain the popularity of English Bulldogs and French Bulldogs, including factors that influenced owners’ decisions to buy brachycephalic dogs [39–41]. The breeds’ appearances (large forehead, big eyes, round face, and bulging cheeks), good behavior, deeply affectionate temperament and good relationships with children have been described as the most important determinants driving people’s desire for these breeds [17,37–39].

All of the typical brachycephalic features valued by the owners of Boxers can be found in English Bulldogs and French Bulldogs; however, the Bulldogs have a breed size more suitable to current lifestyles. The large size of Boxers could somewhat influence buyers’ breed choice and may explain the decline of this breed in our clinical setting.

PS in American Staffordshire Terriers and Golden Retrievers progressively increased from 1997 to 2017; this observation is in contrast with our explanation for the decrease in Boxer popularity regarding their size. In fact, even if the breeds are medium or large in size, they are very different from Boxers, not interchangeable, and their success was because they became fashionable. For example, American Staffordshire Terriers, are a status symbol among some young people groups’ in large European cities, and the success of Golden Retrievers was due more to the influence of movies (Fig 3) [19].

Since 1963 (The Incredible Journey—Walt Disney) to 2017 (A Dog’s Purpose—Lasse Hallström), Golden Retrievers have been movie stars, which is a well-known reason to explain the increasing popularity of the breed in a social context, and many studies have been performed to explain how media can influence a buyer’s choice [17–19].

PDA was the second most common CHD in our population, in both pure and crossbreed dogs. The presence of PDA was significant in females; therefore, a penetrant autosomal recessive and sex-linked inheritance can be excluded [30]. Although PDA was absent in Boxers, our results indicate the prevalence of PDA was higher than in studies performed in United States and Europe [4–7]. The reason for the change in the frequency of PDA we observed from 2006 to 2011 in that period of time could be explained by the use of Amplatzer Canine Duct Occluder, which is suitable for large breed dogs and that became available in our center in 2006 (Fig 4) [12,13,32,33,42–44].

Nine cases of rPDA were found in Maltese; it is a very uncommon CHD, and in this breed has only been described in a publication issued from the same RC in 2011 [16].

It is interesting to note trend of SAS, it was the 2^nd^ most common CHD in our population from 1997 to 2011, and then its frequency decreased through 2017.

SAS and PS are very commonly associated with each other in Boxers (85.8%), and the screening program at this center was aimed to reduce the incidence of both (Fig 4). The reduction of SAS in Boxers is an interesting result because it demonstrates the effectiveness of the screening and breeding program in Boxers. In other words, the increased prevalence of PS in 20 years is not a failure of the Boxer screening and breeding program, but rather the result of the large increase in fashion breeds, such as the French Bulldog, English Bulldog and American Staffordshire Terrier, that are not screened [15,20]. SAS was also common in our population of Dogue the Bordeaux, compared with the other breeds (Tab.2) [42,43].

The dogs diagnosed with AS were older than the dogs affected by other CHD_s_, and the extreme ages at presentation were 50 months (AS) and less than 12 months (TOF) (Fig 1). This result is not surprising because defects of greater severity are associated with the worst symptoms at an early age. Indeed, in many cases, AS is mild in young dogs and becomes progressively worse with age. The murmur in AS can be very soft and necessitate Doppler echocardiographic examination for definitive diagnosis, which is a very different clinical scenario from TOF [12,41].

Many complex defects were found in our population, and PS was the most common CHD detected in association with the other CHD_s_ (SAS, VSD, PDA) (Table 2). The overall prevalence of the PS-SAS association in Boxers (85.8%) seems very high; however, the value has been estimated over the 20-year period (Fig 5). SAS-PDA was very common in Newfoundland; this complex CHD was found in the 84.6% of the admitted dogs belonging to this breed (Fig 5).

PDA and SAS are also very common as simple defects in Newfoundland (Table 3), and the detection of one CHD should be cause for investigation of the other CHD_s_, in order to exclude the presence of both. The left ventricle volume overload due to a large PDA could cause the overestimation of the severity of SAS. In this case, the correction of PDA determines the reduction of the volume overload, and because the gradient across the aortic valve decreases significantly, the actual severity of Subaortic Stenosis can be evaluated only after the ductal closure.

Knowing the association among simple CHD_s_ and the breeds involved could be a useful diagnostic tool that should be taken into account in clinical practice.

From 1997 to 2017, several changes have occurred in the clinical and diagnostic approaches to CHD_s_. The evolution of diagnostic technology, the changing criteria in the classification of some congenital heart diseases, and the increased attention to the selection of breeds prone to CHD_s_ has modified the epidemiological conditions of CHD_s_ in the study RC.

In the final section of the study, the relationships among the popularity, volatility and number of CHD_s_ in individual breeds were investigated over a 20 years’ time period.

The analyses of the popularity of the breeds found that the number of CHD_s_ detected in a breed increases with the number of registrants of that breed in the IKC database. This result can be explained by the response to a growing market demand. In this case, the objective of some breeders is to increase the number of puppies of the breed, and little attention is paid to the gene pool strength, to the selection of the ascendants and to a trustworthy breeding program.

Recent studies indicate that breeds with more inherited disorders have become more popular, not less popular, suggesting that health considerations have been secondary in people’s decision to acquire a specific breed of dog [18,21].

Volatility is the average absolute annual change in IKC registration of dogs belonging to a breed, and it was found to be independent from some breed features (e.g., longer life, inherited genetic disorders, health problems). Societal influences (fashions and fads) have been described as having a primary effect on the popularity of companion breeds, and the volatility of the breeds is an interesting parameter to measure the change in breed popularity over time. The volatility of the French Bulldog was very high (v_i_ = 10), and the OR of disease was also high (6.98 CI 5.60–8.71). In contrast, the volatility of German Shepherds was very low (v_i_ = -3) even though the number of registrations of German Shepard puppies is the highest in 20 years among the breeds in our study population. This observation is in accordance with the results obtained by other authors that found that social influence has been more important than functional traits (e.g., health and trainability) in determining owners’ choice of a breed [18,36,39].

Breed size is thought to be a very important trait behind the owner’s decision to choose a breed. This observation is well supported by the volatility values, with significantly lower or more negative values for large breed dogs, and significantly more positive values for small or medium breed dogs. Chihuahuas, French Bulldogs and CKCS were the most valued small breeds. Among medium size breeds, the Border Collie and American Staffordshire Terrier showed the highest volatility.

However, size was not the only parameter that influenced the popularity of a breed; Yorkshire Terriers and Maltese showed a very low volatility despite their small size.

The influence of media, including movies, television and radio, on the audience is well known and described [17–19]. Unfortunately, people may choose dog breeds based on this media influence and on the idea that a breed is fashionable or a status symbol. These dog owners may not care about the social context in which it should be introduced or the health problems from which a breed may suffer [17,19].

The limitations of this study were primarily associated with its retrospective nature; some cases could not be included because of a lack of clinical and diagnostic information. In particular, the absence of information about the prevalent breeds in crossbreed dog has been a limitation in identifying relationships between breed and CHD in this class of dogs. A bias could also arise because the study was conducted in a single cardiological RC that specializes in the surgical or percutaneous repair of PS, PDA, VSD and ASD, and this center has been unique in our country for a long period of time. The specialization of this RC is the reason why CHD and some breeds (e.g., Boxer, German Shepherd) are overrepresented in our study population. However, this specialization could also be a point of strength because any variation in the preferences of breeds can be monitored from a consistent study location.

## Conclusions

In conclusion, this study allowed us to evaluate the Boxer screening program for CHD_s_, whose success is evidenced by the decreased prevalence of SAS and PS in this breed.

However, the paradox that people buy breeds of dog that are predisposed to congenital heart diseases was also evidenced in our study, and, as reported elsewhere, fashions and trends influence many individual choices [18,19,36,37]. The owners are not often fully aware of the potential problems their dog may face prior to acquisition of a dog [38,45]. It is also possible that owners do not perceive the clinical signs of some inherited cardiac disorders as problems, but rather as normal, breed-specific characteristics (e.g., murmur in CKCS).

In general, when choosing a breed, owners may consider other characteristics to be more important than dog health. Nevertheless, the authors think that an effective breeding program should start with educating the owners about the health problems of a breed. If the owners are not motivated to buy a healthy breed, then breeds with inherent health problems will be perpetuated, and the motivation of breeders to address health problems in their breed reduced.

In this context, the importance of creating a network of veterinary cardiology centers that monitor the distribution of a breed and treat the problem of CHD_s_ using the same clinical approach and diagnostic procedures is clear. This approach could be a useful instrument to provide breeders with effective support in implementing the breeding program in order to control the diffusion of CHD_s_, without impoverishing the genetic pool.
